# Identification of Changes in Wheat (*Triticum aestivum* L.) Seeds Proteome in Response to Anti–*trx s* Gene

**DOI:** 10.1371/journal.pone.0022255

**Published:** 2011-07-19

**Authors:** Hongxiang Guo, Huizhen Zhang, Yongchun Li, Jiangping Ren, Xiang Wang, Hongbin Niu, Jun Yin

**Affiliations:** 1 National Engineering Research Center for Wheat, Henan Agricultural University, Zhengzhou, China; 2 College of Life Sciences, Henan Agricultural University, Zhengzhou, China; 3 College of Public Health, Zhengzhou University, Zhengzhou, China; University of South Florida, United States of America

## Abstract

**Background:**

Thioredoxin h (trx h) is closely related to germination of cereal seeds. The cDNA sequences of the *thioredoxin s* (*trx s*) gene from *Phalaris coerulescens* and the *thioredoxin h* (*trx h*) gene from wheat are highly homologous, and their expression products have similar biological functions. Transgenic wheat had been formed after the antisense *trx s* was transferred into wheat, and it had been certified that the expression of trx h decreased in transgenic wheat, and transgenic wheat has high resistance to pre-harvest sprouting.

**Methodology/Principal Findings:**

Through analyzing the differential proteome of wheat seeds between transgenic wheat and wild type wheat, the mechanism of transgenic wheat seeds having high resistance to pre-harvest sprouting was studied in the present work. There were 36 differential proteins which had been identified by matrix-assisted laser desorption ionization-time of flight mass spectrometry (MALDI-TOF-MS). All these differential proteins are involved in regulation of carbohydrates, esters, nucleic acid, proteins and energy metabolism, and biological stress. The quantitative real time PCR results of some differential proteins, such as trx h, heat shock protein 70, α-amylase, β-amylase, glucose-6-phosphate isomerase, 14-3-3 protein, S3-RNase, glyceraldehyde-3-phosphate dehydrogenase, and WRKY transcription factor 6, represented good correlation between transcripts and proteins. The biological functions of many differential proteins are consistent with the proposed role of trx h in wheat seeds.

**Conclusions/Significance:**

A possible model for the role of trx h in wheat seeds germination was proposed in this paper. These results will not only play an important role in clarifying the mechanism that transgenic wheat has high resistance to pre-harvest sprouting, but also provide further evidence for the role of trx h in germination of wheat seeds.

## Introduction

Pre-harvest sprouting (PHS) is a major limitation to stability of wheat production in parts of the world where long range rainfall or damp conditions prior to harvest often occur. Therefore, there is a need to breed for increased resistance to PHS. Molecular breeding may be an effective method to reduce PHS susceptibility. Thioredoxin h (trx h), a protein catalyzing thiol-disulfide interchange, is involved in the regulation of redox environment of the cell. Thioredoxin h is widely found in many kinds of plants, and was found in wheat seeds by Suske [1]. The *trx s* (*thioredoxin s*) gene from *Phalaris coerulescens* and the *trx h* (*thioredoxin h*) gene from wheat belong to the thioredoxin gene family. Their cDNA sequences are highly homologous, and their expression products have similar biological functions [2]–[4]. Transgenic wheat was formed after the antisense *trx s* was transferred into wheat with particle bombardment. It has been shown that the expression level and content of thioredoxin h in the transgenic wheat seeds is lower than that in wild type [5]–[6], and that the transgenic wheat has lower PHS susceptibility [7].

Much work about the biological functions of thioredoxin h has been reported. Some researchers concluded that thioredoxin h played an important role in the germination of cereals (wheat and barley) by reducing the intramolecular disulfide bonds of storage proteins and other proteins in the starchy endosperm. During seed germination, thioredoxin h can increase the susceptibility of storage proteins to proteolysis by breaking the intramolecular disulfide bonds of storage, and can also change the activity of enzymes either directly by reduction or indirectly by counteracting the inhibitive effect of the inhibitor proteins [8]–[14]. Recently, some study on the transgenic wheat with the antisense trx s has been done [15]–[18], but it is not enough to clarify the mechanism of transgenic wheat seeds having high resistance to PHS.

The classical two-dimensional electrophoresis (2-DE) based proteomic method provides a visual output for protein profiling and comparative mapping of expressed proteins between biological samples. 2-DE in conjunction with mass spectrometry (MS) analysis has been used as the core method for comparative quantitative proteomic studies in many different species, including investigating proteome-level differences between control cell and transgenic cell. The proteomic approach may offer the possibility to identify proteins associated with a particular biological process or response to environment, and it is helpful to clarify these mechanisms on the protein level [19]–[23].

In the present work, to clarify the mechanism of transgenic wheat seeds having high resistance to PHS, the changes in protein profile of wheat seeds were identified by comparative proteomics between wild type and transgenic wheat seeds. 20 differential protein spots in mature seeds and 16 differential protein spots in imbibed seeds were successfully identified by MALDI-TOF-MS. The abundant changes in these identified proteins, as well as their putative functions, are consistent with the proposed role of trx h in wheat seeds. This is the first proteomic study of the wheat seeds in response to trx h, and the results greatly expand our knowledge about the role of thioredoxin h in germination of wheat seeds. A possible model for the role of trx h in wheat seeds germination was proposed in this paper.

## Materials and Methods

### Plant materials

Transgenic wheat seeds with antisense trx s gene and wild type wheat seeds (Yumai 18) were harvested after mature and at 30 days after anthesis respectively, frozen in liquid nitrogen and stored at −80°C. After being sterilized, wheat mature seeds were allowed to germinate at room temperature on sterile filter papers soaked with water for 2 days [2]. Wheat seeds were collected on the second day after imbibition.

### Total soluble protein extraction and quantification

Proteins were extracted using the trichloroacetic acid (TCA)/acetone/2-mercaptoethanol precipitation method developed by Damerval et al. [24], applying some modifications. Wheat seeds were frozen in liquid N_2_ and ground to a fine powder using a ceramic mortar and pestle. One gram of the resulting powder was suspended in 4 ml of chilled (4°C) extraction buffer containing 50 mmol/L tris (hydroxymethyl aminomethane) hydrochloride (pH 7.8), 100 mmol/L KCl, 5 mmol L^−1^ EDTA, 0.70 ml/L 2-mercaptoethanol and 10 ml/L plant protease inhibitor mix (GE Healthcare, Sweden). After centrifuged at 1000 g at 4°C for 15 min, the supernatant was collected and mixed with 20 ml of cold (−20°C) acetone containing 100 g/L TCA and 0.70 ml/L 2-mercaptoethanol, and was maintained at −20°C for at least 1 h to allow protein precipitation. Then, precipitated proteins were centrifuged at 12,000 g at 4°C for 15 min, and the pellet was washed twice in the cold water/acetone solution(20∶80,v/v) containing 0.70 ml/L 2-mercaptoethanol and was centrifuged at 12,000 g for 15 min. After the supernatant was removed, the pellet was air dried and then resuspended in the solution consisting of 9 mol/L urea, 20 g/L 3-[(3-cholamidopropyl) dimethylammonio]-1-propanesulphonate, 30 g/L DTT and 20 ml/L pH 3–10 ampholytes. Finally, the sample was centrifuged at 12,000 g at 4°C for 30 min and the supernatant was subjected to IEF. Proteins were quantified using the 2D QUANT kit (Amersham Biosciences, Sweden) with bovine serum albumin as the standard.

### Two-dimensional electrophoresis

2-DE of proteins was performed in accordance with the following method. Briefly, 450 µl of solution containing 500 µg of proteins was loaded into an immobilized strip (24 cm, pH 3–10) and the strips were covered with paraffin oil. IEF was carried out with an Ettan IPGphor IEF system (Amersham Pharmcia) applying the following conditions. For the rehydration step the voltage was maintained for 12 h at 50 V, then the proteins were focused for 1 h at 500 V, 1 h at 1000 V and 8 h at 8,000 V. The temperature was maintained at 17°C and the current was 75 µA per strip. After IEF, the strips were equilibrated for 15 minutes in equilibration buffer I (50 mM Tris-HCl pH 8.8, 6 M urea, 30% glycerol, 2% SDS and 0.1% DTT) and buffer II (50 mM Tris-HCl pH 8.8, 6 M urea, 30% glycerol, 2% SDS and 0.25% iodoacetamide). The second dimension electrophoresis was conducted according to the method of Laemmli on a 12.5% SDS polyacrylamide gel using an Ettan DALT II System (Amersham Pharmcia) [25]. The electrophoresis was carried out at 25°C and 5 mA per gel until the samples departed from the strips and then 25 mA per gel until the bromophenol blue dye front arrived at the bottom of the gels. Following SDS polyacrylamide gel electrophoresis (SDS-PAGE), gels were stained with Coomassie R-250. A total of six gels were analyzed, three gels for control wheat seeds and three gels for transgenic wheat seeds.

### Gel image and data analysis

The 2-DE image and statistical analysis was performed by using the ImageScanner II (Amersham Pharmcia) and PDQest7.0 software (Bio-Rad). Gel patterns from each group of seeds were matched together and the relative abundances of each spot in the two gel sets (control and transgenic) were compared. The differences in expression between control and transgenic samples were analyzed by Student's t test, and the protein spots showing a significant difference (p<0.05) of abundance change (up or down) were considered as “significantly differential proteins”.

### Spot excision and protein in-gel digestion

The differential protein spots were manually cut out from the gels. Tryptic in-gel digestion was based on the following procedure. Briefly, each gel piece was destained with 25 mM ammonium bicarbonate in 50% acetonitrile till the gels were changed opaque and colorless. Proteins were reduced with 10 mM DTT in 100 mM NH_4_HCO_3_ for 1 h at 60°C and alkylated with 40 mM iodoacetamide in 100 mM NH_4_HCO_3_ for 30 min at room temperature in the dark. The gel pieces were minced and lyophilized, then rehydrated in 25 mM NH_4_HCO_3_ with 10 ng of sequencing-grade modified trypsin at 37°C overnight. After digestion, the peptides were collected and the pellets were washed with 0.1% trifluoroacetic acid in 50% (v/v) acetonitrile three times to collect the remaining peptides.

### Matrix-assisted laser desorption/ionization time of flight MS analysis and database searching

The significantly differential proteins were identified by MALDI-TOF-MS. Mass spectra were acquired using a Voyager-DE STR™ mass spectrometer (Applied Biosystems, USA), and peptides were detected in the reflection-delayed extraction mode. Parameters were set as follows: nitrogen laser (337 nm, 0.5 ns pulse width, 20 Hz repetition rate), delayed ion extraction 100 ns, Grid voltage 70%, vacuum degree 4e-008, single scan of MS signals accumulating 200 times, positive spectra determination.

Sequence similarity searches based on MALDI-TOF-MS/peptide mass fingerprinting (PMF) were performed with Mascot software (Matrixscience Company) using default parameters against NCBInr Database.

### Quantitative RT-PCR analysis

Total RNA was isolated using TRIzol reagent (Invitrogen, CA, USA) to extract the RNA from 100 mg of transgenic wheat and wild type seeds at 2 days after germination. First-strand cDNA was synthesized from 2 ug of total RNA using the M-MLV Reverse Transcriptase (Promega, WI, USA) according to the manufacturer's instructions. After reverse transcription, the products of each reaction were diluted 5 times to avoid potential primer interference in the following qRT-PCR reaction.

Quantitative real time PCR was performed using the TaKaRa SYBR® Premix Ex Taq™ II (Perfect Real Time) on a Bio-Rad IQ5 Real-Time PCR Detection System. The volume of qRT-PCR reaction was 25 ul, and tubulin was used as the endogenous reference gene. Each reaction consisted of 2 µl of product from the diluted reverse transcription reaction, 0.5 µl of primers (forward and reverse), 12.5 µl of 2×SYBR® Premix Ex Taq™ (2×), and 9.5 µl of nuclease-free water. The reactions were incubated in a 96-well plate at 95°C for 30 s, followed by 40 cycles of 95°C for 5 s, 57°C for 30 s and 72°C for 30 s. All reactions were run in three replicates for each sample. The primer pairs used for quantitative RT-PCR are shown in Table 1, and they were designed based on wheat EST sequences of candidate proteins available in the NCBI.

**Table 1:** Sequences of the primers used for real-time PCR.

Gene	Primer sequence
Thioredoxin h	F-5′-AGGACAGGGTTGTGGGAGCT-3′
Thioredoxin h	R-5′-AGAGGCACCGCTTCTTGAGC-3′
Serpin	F-5′-CGGTCTCCCTCTACTCCGCA-3′
Serpin	R-5′-GACCTTGCCTGTCCCGAGTG-3′
HSP70	F-5′-AAGGAGCTCGGGGACAAGGT-3′
HSP70	R-5′-TGTCGATCCACCAGCAACGG-3′
β-amlyase	F-5′-ATCCGGCCCAGTACCATCGG-3′
β-amlyase	R-5′-TCCGTCTCGGGATCGAACGG-3′
14-3-3	F-5′-CCTGACGCTGTGGACTTCCG-3′
14-3-3	R-5′-TCCAGATTCACCCTTGGGAGCA-3′
WRKY6	F-5′-CGGGCCAGTTTGCAATGAGC-3′
WRKY6	R-5′-TGTGGAAATGGACTCGGCAGC-3′
glyceraldehyde 3-phosphate dehydrogenase (GAPDH)	F-5′-GGCCGGGATTGCTCTGAACG-3′
glyceraldehyde 3-phosphate dehydrogenase (GAPDH )	R-5′-TGGTGCTGTGCATGTGACGG-3′
glucose-6-phosphate isomerase (GPI)	F-5′-GAGCTGGGCAAGTCACTGGC-3′
glucose-6-phosphate isomerase (GPI)	R-5′-TTGCGCTGCTGGGGTTGAAG-3′
S3-RNase	F-5′-TCGTCCACGGTGGGATACGA-3′
S3-RNase	R-5′-AGGCCACAAACCGTGAACCG-3′
α-amylase	F-5′-AAGGTCATGCAGGGCTACGC-3′
α-amylase	R-5′-CGAACACATGGTCGTAGAAGATGC-3′
Tubulin	F-5′-GGTGCTTACCGCCAGCTCTT-3′
Tubulin	R-5′-TGGTGTAATGACCACGGGCG-3′

## Results and Discussion

### The comparative analysis of 2-DE maps of wild type and transgenic wheat seeds

**Figure 1:**
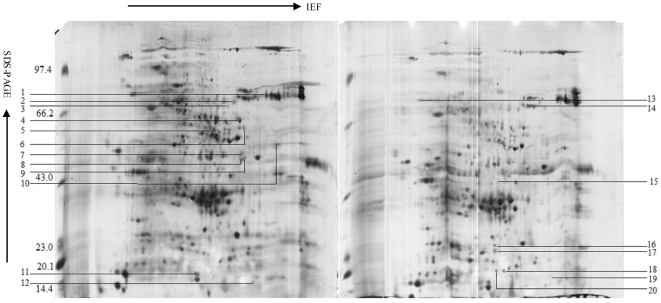
2DE of mature wheat seeds. The left figure: Yumai 18; The right figure: Transgenic Yumai 18.

**Figure 2:**
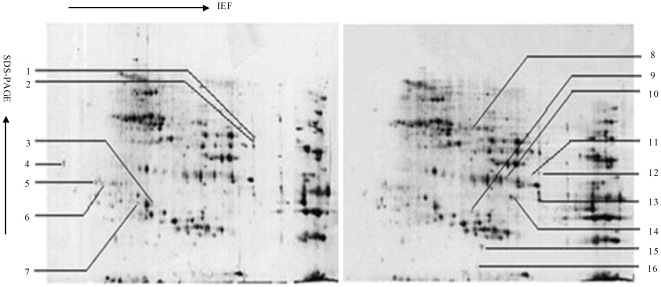
2DE of wheat seeds imbibed for 2 d. The left figure: Transgenic Yumai 18; The right figure: Yumai 18.

In order to investigate the changes of wheat seed proteome in response to antisense trx s gene, 2-DE analysis of the total proteins in wild type and transgenic wheat seeds in different stages was carried out. The 2-DE electrophoretic maps obtained from wild type mature seeds and transgenic mature seeds were showed in Figure 1, and the 2-DE electrophoretic maps obtained from wild type imbibed seeds and transgenic imbibed seeds were showed in Figure 2. The protein spots showed a broad distribution in the pI range from 3.0 to 10.0 and the mass range from 10 to 120 kDa. Approximately 850 protein spots were detected on Coomassie R-250 stained gels and about 600 protein spots were matched between three control gels and three treated gels. Spot intensity variations between wild type and transgenic samples were quantified by software image analysis, and the protein spots showing significant differences (p<0.05) were selected. 20 proteins were selected as “significantly differential proteins” in Figure 1, including 12 down-regulated and 8 up-regulated proteins in transgenic wheat seeds. 16 proteins were considered as “significantly differential proteins” in Figure 2, including 9 down-regulated and 7 up-regulated proteins in transgenic imbibed wheat seeds.

### The identification of differential proteins

These significantly differential proteins were analyzed by MALDI-TOF-MS. By searching protein database, these significantly differential proteins were identified according to pI and molecular weight (Tables 2 and 3). Some of the identified proteins have been well characterized in terms of their response to antisense trx s gene, and the role of other proteins is not clear yet in relation to trx s gene. As shown in Table 4, there were 11 differential proteins involved in protein metabolism, including Serpin (serine proteinase inhibitors), protein disulfide isomerase (PDI), Heat shock protein 70 (Hsp70), BIP, small subunit ribosomal protein, elongation factor 1 alpha, phenylalanyl-tRNA synthetase, NADH glutamate synthase, glutamate dehydrogenase (GDH), aspartate aminotransferase and adenylylsulfate kinase (APK). Serpin and PDI have been proved to be related to degradation of the seeds storage proteins, and other proteins are related to proteins biosynthesis during the germination of wheat seeds. 4 differential proteins involved in saccharide metabolism were indentified. XIP-I may have a function in inhibiting amylase, and phosphoenolpyruvate carboxylase (PEPC), glyceraldehyde-3-phosphate dehydrogenase (GAPDH) and glucose-6-phosphate isomerase (GPI) are enzymes related to starch degradation. There were 3 differential proteins related to gene expression (WRKY transcription factor 6, 14-3-3 protein and maturase K) and 5 differential proteins related to the resistance (Puroindoline b, alcohol dehydrogenase, disease resistance protein OB5, catalase isozymes, and Glutathione transferase). There were also 5 differential proteins (RPP13, S3-RNase, plasmalemma H^+^-ATPase, acid phosphatase and 7S storage globulins) related to other functions. After the antisense trx s gene was transferred, the germinating speed of transgenic wheat seeds was slowed [2]. Therefore, it is not surprising to find these differential proteins.

**Table 2:** The identification of the differential proteins in mature seeds.

Spot number	Accession number	Number of matched peptide	Protein identification	PI	Mr (KD)	Change fold
1	gi|15954	7/40	S3-RNase	9.28	16.0	−6.1
2	gi|50902006	10/31	aspartate aminotransferase	8.61	49.9	−5.6
3	gi|50902006	14/36	aspartate aminotransferase	8.61	49.9	−7.2
4	gi|806595	11/50	glutamate dehydrogenase	6.28	44.5	−11.0
5	gi|46394266	12/54	WRKY transcription factor 6	9.45	39.8	−9.4
6	gi|3928886	11/19	protein disulfide isomerase(PDI)	5.29	25.6	−10.5
7	gi|18978	10/25	glyceraldehyde 3-phosphate dehydrogenase	6.67	36.5	−5.4
8	gi|73622088	14/47	xylanase inhibitor protein I precursor **(XIP-I)**	8.66	33.3	−5.1
9	gi|73622088	15/41	xylanase inhibitor protein I precursor **(XIP-I)**	8.66	33.3	−8.1
10	gi|73622088	18/69	xylanase inhibitor protein I precursor **(XIP-I)**	8.66	33.3	−5.7
11	gi|7229451	17/35	RPP13	6.08	97.1	−6.8
12	gi|13604161	8/13	plasmalemma H^+^-ATPase	6.74	35.7	−8.9
13	gi|303503	17/123	PEPC	5.14	62.7	+5.7
14	gi|3329471	6/22	5′-adenylylsulfate kinase	9.31	30.1	+6.3
15	gi|1345673	13/51	Catalase isozyme 1	6.97	57.0	+5.0
16	gi|1070354	7/24	Hv14-3-3b	4.67	29.7	+11.2
17	gi|13937096	7/18	disease resistance protein OB5	9.68	18.7	+12.3
18	gi|32348726	6/15	small subunit ribosomal protein	10.17	9.7	+11.1
19	gi|55056888	5/6	ATP synthase beta subunit	5.3	48.5	+5.1
20	gi|13235635	11/49	puroindoline b	8.83	16.7	+11.6

Change Fold: + means increase in transgenic wheat, − means decrease in transgenic wheat.

**Table 3:** The identification of the differential proteins in seeds imbibed for 2 d.

Spot number	Accession number	Number of matched peptide	Protein identification	PI	Mr (KD)	Change Fold
1	gi|50946885	10/36	glucose-6-phosphate isomerase precursor (GPI)	6.42	67.2	+6.1
2	gi|50946885	11/34	glucose-6-phosphate isomerase precursor (GPI)	6.42	67.2	+6.3
3	gi|59938926	11/35	maturase K-like protein	10.5	52.7	+5.1
4	gi|20197523	5/16	Serpin	8.6	37.1	+8.9
5	gi|6684367	5/25	alcohol dehydrogenase	5.84	41.3	+7.8
6	gi|6016173	7/80	glutathione transferase omega 1	6.23	27.5	+7.1
7	gi|31321944	9/38	vicilin seed storage protein	6.38	55.6	+7.7
8	gi|50902006	12/32	aspartate aminotransferase	8.61	49.9	−7.5
9	gi|62177685	11/20	NADH glutamate synthase	6.18	24.0	−7.9
10	gi|O95363	11/22	Phenylalanyl-tRNA synthetase	5.31	52.3	−6.8
11	gi|18405204	5/18	acid phosphatase	5.71	32.7	−5.3
12	gi|73622088	16/50	xylanase inhibitor protein I precursor (XIP-I)	8.66	33.3	−7.8
13	gi|30693966	8/26	BIP	5.18	67.4	−5.8
14	gi|87241037	10/20	Heat shock protein Hsp70	5.11	71.0	−6.5
15	gi|40748293	5/10	elongation factor 1 alpha	8.47	26.5	−6.3
16	gi|93211071	13/35	RPP13 variant	6.54	96.1	−5.1

Change Fold: + means increase in transgenic wheat, − means decrease in transgenic wheat.

**Table 4:** Changes in wheat seeds proteins induced by anti-trx s gene.

protein	changes	function
		**Proteins metabolism**
PDI	↑	Help proteins fold(forming disulfides)
serpin	↑	Inhibit serine proteinase
glutamate dehydrogenase	↓	Participate amino acid metabolism
aspartate aminotransferase	↓	Participate amino acid metabolism
adenylylsulfate kinase	↓	Assimilate S and transform into Cys and Met
BIP	↓	Combining ATP and act as molecular partner
small subunit ribosomal protein	↓	Participate the synthesis of proteins
NADH glutamate synthase	↓	Catalyze the synthesis of Glu
Phenylalanyl-tRNA synthetase	↓	Transport Ala during the synthesis of proteins
elongation factor 1 alpha	↓	Participate the synthesis of proteins
Heat shock protein Hsp70	↓	Act as molecular partner
		**Saccharide metabolism**
XIP-I	↑	Inhibit the amylase
GAPDH	↓	Participate glucolysis
glucose-6-phosphate isomerase	↓	Participate glucolysis
PEPC	↓	Supplement intermediate product for glucolysis in seeds germination
		**Gene expression**
WRKY transcription factor 6	↓	Regulate the expression of storage proteins, α-, β-amylase
maturase K-like protein	↓	Participate the processing after transcription
14-3-3 proteins	↓	Participate transcription, primary metabolism and regulate plasmalemma H^+^-ATPase, nitric acid reductase and sucrose cleaving enzymes
		**Nucleic acid metabolism**
S3-RNase	↑	Decompose RNA
		**Resistance**
Catalase isozyme 1	↑	Anti-oxidant enzyme
alcohol dehydrogenase	↑	Participate anaerobic metabolism in plant
puroindoline b	↑	Regulate seeds hardness
disease resistance protein OB5	↑	Related to resistance
Glutathione transferase	↑	Related to resistance
		**Lipoid metabolism**
acid phosphatase	↓	Decompose lipoid
		**Energy metabolism**
ATP synthase	↓	Decompose ATP and release energy
		**Other metabolism**
plasmalemma H+-ATPase	↓	Acidify cell wall and induce water uptake of seeds

↓ represents down-regulation; ↑ represents up-regulation.

### Transcript analysis of differential proteins

**Figure 3:**
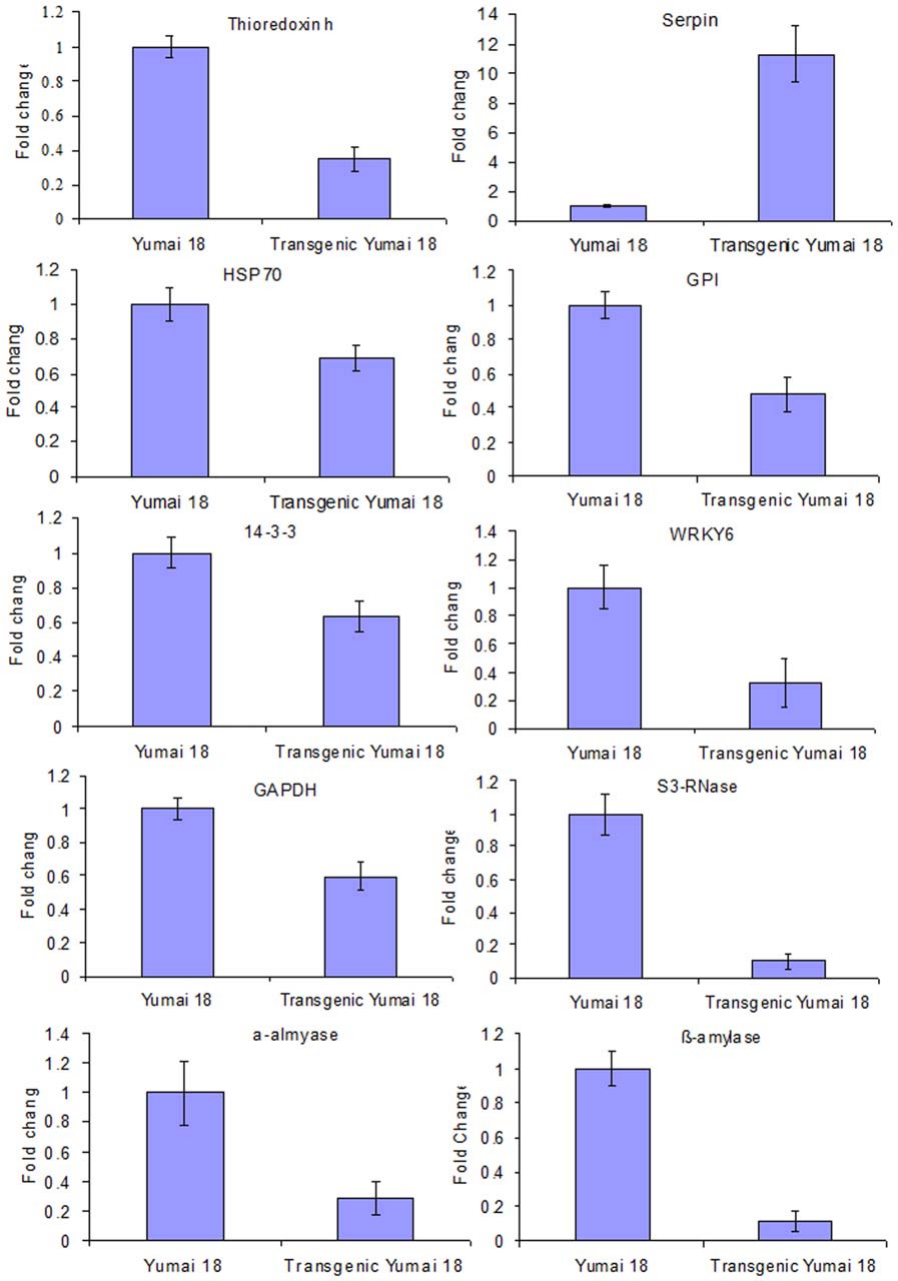
Changes in the relative abundance of selected transcripts in response to anti-trx s gene. Fold change was determined using the 2^−ΔΔCt^ method and error bars represent the standard deviation of the mean (SD).

RNA extracted from wheat seeds germinated for 2-day was used to verify the changed expression in transcriptional level and evaluate the correlation between mRNA and protein levels. qRT-PCR analysis for ten selected transcripts including trx h, Serpin, HSP70, α-amylase, β-amylase, GPI, 14-3-3 protein, S3-RNase, GAPDH, and WRKY transcription factor 6 are shown in Figure 3. Since gene expression is controlled at multiple levels, it is necessary that the transcript level and protein abundance may not exactly correlate. In our study, the transcript levels of trx h, HSP70, α-amylase, β-amylase, GPI, 14-3-3 protein, S3-RNase, GAPDH, and WRKY transcription factor 6 were significantly lower in transgenic wheat, while the Serpin level was significantly greater in transgenic wheat, which represented good correlation between transcripts and proteins.

### The prediction of disulfide bonding state of some differential proteins

**Figure 4:**
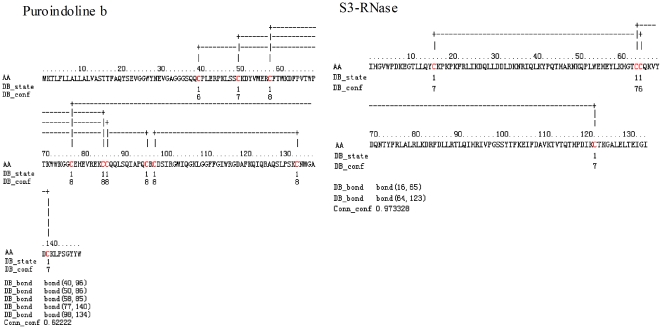
The predicting results of disulfide bonding state of some differential proteins. AA, amino acid sequence; DB_state, predicted disulfide bonding state (1 = disulfide bonded, 0 = not disulfide bonded); DB_conf, confidence of disulfide bonding state prediction (0 = low to 9 = high); DB_bond, position in sequence of a pair of cysteines predicted to be forming a disulfide bridge; Conn_conf, confidence of connectivity assignment given the predicted disulfide bonding state (real value in [0,1]).

Disulfide bridges play a major role in the stabilization of the folding process for some proteins. Prediction of disulfide bridges from sequence alone is therefore useful for the study of structural and functional properties of specific proteins. In addition, knowledge about the disulfide bonding state of cysteines may help the experimental structure determination process and may be useful in other genomic annotation tasks. In this study, the disulfide bonding state of two differential proteins (Puroindoline b and S3-RNase) were predicted with Cysteines Disulfide Bonding State and Connectivity Predictor (http://disulfind.dsi.unifi.it/) [26]. As shown in Figure 4, Puroindoline b and S3-RNase contain 5 and 2 intrachain disulfide bridges, respectively. Therefore, the structural and functional properties of puroindoline b and S3-RNase might be affected directly by trx h.

### The differential proteins involved in protein metabolism

There are 11 differential proteins involved in protein metabolism. Thiocalsin catalyzes the degradation of the seeds storage proteins which have rich disulfides, after these proteins have been reduced during seeds germination [27]. Serpin is a specific inhibitor of thiocalsin and the high abundance of Serpin in transgenic wheat seeds induces the low activity of thiocalsin, so the degradation of the seeds storage proteins is slowed [16]. PDI can catalyze the forming of disulfide and serve as molecular chaperones helping proteins fold and activating inhibitors of alkaline proteases. As a target of thioredoxin, PDI will be deactivated after it is reduced by thioredoxin. Since the quantity of trx h in transgenic wheat is lower than that in wild type wheat, the activity of PDI in transgenic wheat is higher than that in wild type wheat. We had proved that the seeds storage proteins have fewer disulfides in transgenic wheat [16]. The other 9 differential proteins are the proteins related to the synthesis of proteins. The expression levels of Hsp70, BIP, Elongation factor 1 alpha, phenylalanyl-tRNA synthetase, GDH, aspartate aminotransferase and NADH glutamate synthase in transgenic wheat are lower than that in wild type wheat. The activitives of GDH, APK and aspartate aminotransferase depend on sulfydryl [28]–[30]. The quantity of trx h in transgenic wheat is lower than that in wild type wheat, so their activities in transgenic wheat are lower than that in wild type wheat. Small subunit ribosomal protein is a component of ribosomal which participates in the protein synthesis. After small subunit ribosomal protein is oxidized into dimmer, it can not participate in the protein synthesis [31]. Because of the lower content of thioredoxin h in transgenic wheat, small subunit ribosomal protein oxidized is more than that in wild type wheat. Therefore, the protein synthesis in transgenic wheat is slower than that in the control during seeds germination.

### The differential proteins involved in saccharide metabolism

There are 4 differential proteins involved in saccharide metabolism. XIP-I is a bifunctional inhibiting protein. XIP-I precursor contains four cysteines, which can form two intermolecular disulfides when XIP-I precursor is transformed into XIP-I. On the other hand, XIP-I can be reduced into XIP-I precursor by some reducing substance, such as trx h. The abundance of the XIP-I precursor in transgenic wheat is lower than wild type wheat. This result is accordance with lower quantity of trx h in transgenic wheat. It had been demonstrated that XIP-I can inhibit the amylase from barley [32]. If XIP-I can inhibit the amylase from wheat, it may play a role in decreasing the degradation of starch during transgenic seeds germination. There are two kind of PEPC (EC 4.1.1.31) isoforms (108 KD and 103 KD) in wheat. PEPC generated in seeds filling stage participates primarily in seeds germination. PEPC can be hydrolyzed by an enzyme containing disulfide. Since the apparent molecular weight of the protein is 62.8 KD, it should be the product of PEPC degradation [33]. The activity of enzyme hydrolyzing PEPC is higher in transgenic wheat than that in wild type wheat because of the lower content of thioredoxin h, so the fewer PEPC participate in transgenic seeds germination. GAPDH, a key enzyme in glycolysis, can be activated by thioredoxin [34]. The quantity of trx h in transgenic wheat is lower than that in wild type wheat, therefore, GAPDH activity in transgenic wheat is lower than that in wild type wheat. GPI (EC 5.3.1.9) is an enzyme participating in the degradation metabolism of saccharide, and its activity depends on sulfydryl [35]. The precursor will be transformed into glucose-6-phosphate isomerase after the disulfide is reduced into sulfydryl. The result in the paper indicates that this transformation has been slowed.

### The differential proteins involved in gene expression regulation

3 differential proteins have relation to gene expression. WRKY transcription factor 6 can recognize the W-box of α-amylase and β-amylase [36]. The abundance of WRKY transcription factor 6 in transgenic wheat is lower than that in wild type wheat, so trx h may have a role in regulating the amylase [15]. 14-3-3 protein has a relation to some physiology metabolism, such as gene transcription, primary metabolism, ion transportation, the activities of enzymes in chloroplast and mitochondria, and so on. The native 14-3-3 protein is a dimmer [37]. The abundance of 14-3-3 protein monomer in transgenic wheat is higher than that in wild type wheat. It had been proved that 14-3-3 protein has a relation to the barley germination [38]. Maturase K, a kind of maturase, participates in gene expression. Maturase K-like protein contains many Cys, but maturase K-like protein having disulfide has no activity [39], which indicates that its activity will be regulated by trx h.

### The differential proteins related to the resistance

There were 5 differential proteins related to the resistance. Puroindoline b has a relation to seeds hardness. It contains rich cysteine and can form five disulfides [40]. It is easier for puroindoline b to form disulfides in transgenic wheat because of the lower content of thioredoxin h, so the hardness of transgenic seeds is higher than the wild type seeds. Glutathione transferase is an enzyme related to resistance. There are two kinds of catalase isozymes in wheat seeds. Catalase is not only an anti-oxidant enzyme, but also an enzyme relating to seeds germination [41]. The putative disease resistance protein OB5 is a protein related to resistance, but there is the fewer report on it. Alcohol dehydrogenase (EC 1.1.1.1), an enzyme involved in the degradation metabolism of saccharide, is related to anaerobic metabolism in plant. During the seeds germination, the activity of alcohol dehydrogenase gradually decreases [42].

### The differential proteins related to other function

Some differential proteins related to other function were also found. S3-RNase, having two disulfides, is a target protein of thioredoxin. After reduced, S3-RNase will be deactivated [43]. The content of thioredoxin h in transgenic wheat is lower than that in wild type wheat, so the fewer S3-RNase is reduced and the activity of S3-RNase is higher in transgenic wheat. It is easy to understand that the proteins are synthesized slowly in transgenic wheat. Plasmalemma H^+^-ATPase is a kind of ATPase transporting H^+^. Some research has reported that the activity of plasmalemma H^+^-ATPase increased during seeds germination [44]. Plasmalemma H^+^-ATPase can promote the acidification of cell wall and induce the further absorbing of cell, accelerating the seeds germination. RPP13 is one of thioredoxin family [45]. Acid phosphatase is an enzyme related to degradation of storage material in seeds. Its activity rises to top at the fourth day after germination, and the rise is the result of gene expression [46]. 7S storage globulins are a kind of seed storage proteins, and it is gradually degraded in seeds germination. The high abundance of 7S storage globulins in the transgenic seeds indicates that its germination is slower than the wild type wheat.

### A possible regulation model for the role of trx h in wheat seeds germination

**Figure 5:**
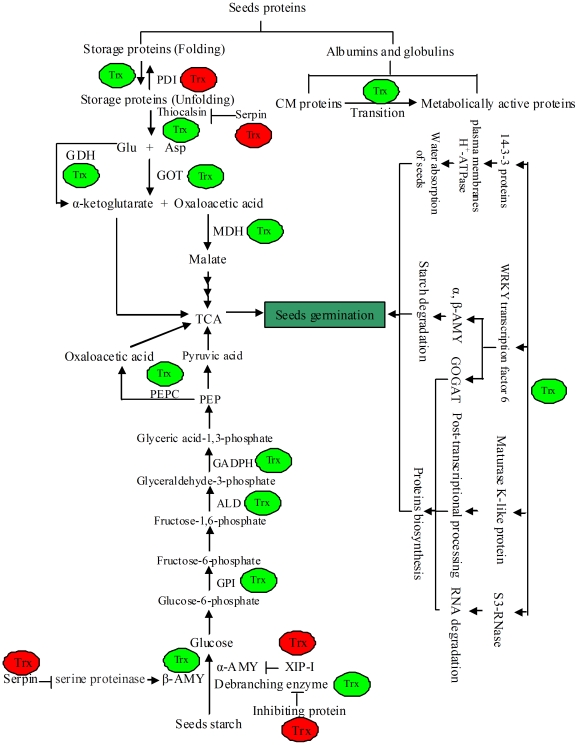
A proposed model for the role of Trx h in wheat seeds germination. Abbreviations are as follows: α-AMY α-amlyase, β-AMY β-amlyase, XIP-I Xylanase inhibitor protein I, GDH glutamate dehydrogenase, GOT aspartate aminotransferase, MDH Malate dehydrogenase, GPI glucose-6-phosphate isomerase, CM proteins chloroform-mwthanol proteins, PEPC phosphoenolpyuvate carboxylase, GAPDH Glyceraldehyde-3-phosphate dehydrogenase, PDI protein disulfide isomerase, ALD aldolase, GOGAT glutamic-oxalacetic transaminease. The green and red respectively mean increasing or decreasing the activity or expression of the targets.

It has been proved that trx h is a central regulatory protein of seeds, and a regulation model is proposed according to the results in this paper (Figure 5). To facilitate seeds germination, trx h acts not only by reducing disulfide groups of diverse seed proteins (storage proteins, enzymes and enzyme inhibitors) to increase the transition from CM proteins to metabolically active proteins but also by regulating some genes expression (Figure 5). During seeds germination, trx h plays an important role in promoting proteins degradation. (1) Trx h can increase the susceptibility of storage proteins to proteolysis by breaking the intramolecular disulfide bonds of storage and inhibiting the activity of PDI [16]. (2) Trx h can increase the activity of Thiocalsin by reducing disulfide groups and inhibiting Serpin [16]. (3)Trx h can increase the activities of GDH, GOT and MDH [16]. Trx h also has a function in accelerating the starch degradation during seeds germination. (1) Trx h can increase the activities of α-AMY, β-AMY and debranching enzyme by reducing disulfide groups of these enzymes or inactivating the inhibiting proteins of these enzymes, such as XIP-I and serpin. (2) The expression level of α-AMY and β-AMY can be increased by trx h. (3) Trx h can increase the activities of PEPC, GAPDH, ALD and GPI.
